# Retinal hemodynamic effects of sub-Tenon anesthesia

**DOI:** 10.3389/fnins.2026.1759889

**Published:** 2026-02-27

**Authors:** Anthia Papazoglou, Tim J. Enz, Ulrike Steitz, Muriel Dysli, Markus Tschopp

**Affiliations:** 1 Department of Ophthalmology, Cantonal Hospital Aarau, Aarau, Switzerland; 2 Department of Ophthalmology, University Hospital Zurich, University of Zurich, Zurich, Switzerland; 3 Department of Ophthalmology, University of Basel, Basel, Switzerland; 4 Department of Ophthalmology, Inselspital, Bern University Hospital and University of Bern, Bern, Switzerland

**Keywords:** cataract surgery, diabetes, intraocular pressure, local anesthesia, mepivacaine, perfusion, retinopathy, sub-Tenon

## Abstract

**Introduction and objectives:**

Sub-Tenon anesthesia techniques are commonly employed in ophthalmology. However, there is speculation that these techniques may have hemodynamic effects on the eye, potentially affecting patients with impaired ocular perfusion. This study aims to evaluate the impact of sub-Tenon anesthesia during cataract surgery on retinal vessel density (VD) and perfusion density (PD) in eyes affected by diabetic maculopathy (DM) or retinal vein occlusion (RVO), using optical coherence tomography angiography (OCTA).

**Methods:**

The study included 20 patients receiving sub-Tenon anesthesia with unpreserved mepivacaine 2% for unilateral cataract surgery: 10 with DM and 10 with RVO, analyzing a total of 40 eyes (20 treated and 20 untreated). Blood pressure, intraocular pressure (IOP), and retinal microvascular changes measured via OCTA (VD, PD) were documented before and shortly after sub-Tenon anesthesia. The contralateral eye, which did not receive anesthesia, served as a control.

**Results:**

A negative association between sub-Tenon anesthesia to VD and PD was observed, with a VD-change of −1.5 ± 2.8 mm/mm^2^ (*p* = 0.02) in the treated eye compared to 0.7 ± 3.4 mm/mm^2^ in the untreated eye (*p* = 0.36) and a PD-change of −3.7 ± 7.0% (*p* = 0.029) in the treated eye compared to 1.6 ± 8.2% in the untreated eye (*p* = 0.38), suggesting a potential hemodynamic effect of sub-Tenon anesthesia. However, the quality of OCTA images strongly influenced these findings. After excluding lower-quality images (signal strength index <6), no statistically significant difference was observed between the intervention and control eyes, indicating a false positive correlation due to image artifacts. In the overall population, no significant changes in blood pressure and intraocular pressure were observed before and after the injection.

**Conclusion:**

This study did not prove retinal microvascular changes associated with sub-Tenon anesthesia. The observed VD and PD reductions were likely caused by OCTA image artifacts rather than true hemodynamic changes.

## Introduction

Sub-Tenon anesthesia for ocular surgery was introduced by Stevens (1992) and is widely used, due to its ease of administration, effectiveness, and safety profile (Adekola et al., 2018). The sub-Tenon’s block is employed in various procedures, including cataract, strabismus, and vitreoretinal surgery. This technique entails the transconjunctival administration of an anesthetic solution into the potential sub-Tenon space with a blunt canula. Its purpose is to deliver effective analgesia and in higher dosage akinesis, while reducing the risks associated with sharp needle techniques, such as globe perforation, retrobulbar hemorrhage, optic nerve injury, and accidental injection into the subarachnoid space (Kumar and Dodds, 2006; Kumar et al., 2005). Research has indicated that it is at least as effective as peribulbar anesthesia (Parkar et al., 2005; Guise, 2012; Antony et al., 2022).

Despite its advantages, concerns have been raised about the potential hemodynamic effects of the sub-Tenon technique on ocular perfusion (Coşkun et al., 2014; Kumar et al., 2011; Fry and Ring, 2008; Feibel and Guyton, 2003). Coşkun et al. (2014) suggested that sub-Tenon anesthesia decreased ocular blood flow significantly by using color doppler imaging. While various studies suggest that the rise in pressure is mainly a result of the volume effect caused by the local anesthetic agent being forcefully injected into the restricted space of the bony orbit (Fry and Ring, 2008). Larger volumes of injectate may lead to a more significant increase in IOP, but also factors such as the orbital volume, the tension in the extraocular muscles, orbital septum, and the conjunctival/Tenons incision acting as a pressure valve, influence this potential rise (Alwitry et al., 2001). Further, it is hypothesized that touching the arterioles with the tip of the blunt cannula could result in a spasm, which would cause vessels to close. This mechanism has been demonstrated by Lende et al. in an experiment with cats (Ellis, 1964). Finally, injecting anesthetic solution into the sub-Tenon space might lead to pharmacological vasoconstriction caused by the high local concentration of the anesthetic drugs (Creese et al., 2017; O’Day et al., 2019; Chen, 2017; Meyer et al., 1993; Watkins et al., 2001). The hypothesis is that the anesthetic drugs either inhibit endothelium-dependent relaxation of the ciliary arteries or attenuate the activity of the vasodilatory nerves (Hulbert et al., 1998). Hereby they alter the blood flow dynamics and decrease the pulsatile ocular blood flow (Rüschen et al., 2003). This may result in a temporary decrease in vessel density (VD) and perfusion density (PD) at the retinal microvascular level.

Due to concerns about retinal ischemia following sub-Tenon anesthesia, some publications have periodically reiterated recommendations to avoid its use in patients with compromised retinal perfusion (Rüschen et al., 2003). According to different authors this concern is especially relevant for patients with diabetic retinopathy or maculopathy (DM), and retinal vein occlusion (RVO) (Creese et al., 2017; Pianka et al., 2001; Enz et al., 2022). These conditions are characterized by impaired autoregulation, endothelial dysfunction, and structural microvascular loss, including capillary non-perfusion, increased retinal vascular permeability, and ischemia-related macular edema (Haydinger et al., 2023). Consequently, the hemodynamic response to sub-Tenon anesthesia may differ from that in eyes without pre-existing vascular disease, with greater pressure dependence and reduced ability to compensate for transient decreases in perfusion (Kohner et al., 1995). Any additional disruption of retinal perfusion could exacerbate pre-existing conditions, potentially leading to adverse visual outcomes (Kohner et al., 1995).

Optical coherence tomography angiography (OCTA) is a relatively new imaging modality that allows non-invasive visualization of the retinal microvasculature. OCTA can provide detailed maps of retinal blood flow and is an excellent tool for assessing retinal microvasculature (Gandhi et al., 2024). It is a non-invasive imaging technique that provides several benefits over conventional angiography methods, such as fluorescein (Kwiterovich et al., 1991) or indocyanine green (Yannuzzi et al., 2012) angiography. Being non-invasive, OCTA does not require the injection of a dye into the patient’s bloodstream, thereby eliminating the risks associated it: anaphylaxis, nausea, vomiting, rash, or skin and urine discoloration (Chua et al., 2024). However, image artifacts, due to movement, blinking, alteration of corneal surface refractive index or other irregularities, can affect the quality and accuracy of the OCTA scans, potentially leading to false-positive findings (Anvari et al., 2021).

This study aims to investigate the effects of sub-Tenon anesthesia on retinal microvasculature in patients with DM or RVO undergoing cataract surgery. By using OCTA, we assessed VD and PD before and after anesthesia, while also considering the impact of image’s quality on the reliability of our results.

## Methods

This prospective, monocentric study included patients suffering from DM or RVO scheduled for cataract surgery at the Cantonal Hospital Aarau, Switzerland, and was approved by the Ethics Committee Northwest and Central Switzerland (EKNZ; approval ID 2019-01689). We excluded eyes with ocular ischemic syndrome; uncontrolled ocular hypertension (baseline IOP > 25 mmHg) or recent IOP-lowering procedures (<4 weeks); carotid artery stenosis; severe or unstable cardiovascular disease; severe or uncontrolled hypertension; hematological disorders affecting viscosity or oxygen-carrying capacity; active uveitis/infection; recent intraocular surgery (<3 months); media opacity other than cataract; or low-quality OCTA imaging. No formal *a priori* sample size calculation was performed because the study was exploratory. All procedures were performed in accordance with the Declaration of Helsinki and applicable national laws and regulations. The study population consisted of 10 patients diagnosed with DM and 10 with RVO.

### Intervention day

**Figure 1:**
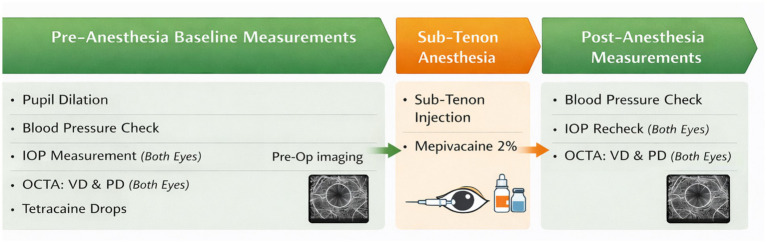
Study timeline.

At the day of planned intervention, the patients included in the study underwent the following measurements in the pre-operative room: bilateral pupil dilation, blood pressure (BP) was recorded using a standard automated cuff, intraocular pressure (IOP) was measured using iCare tonometry (iCare Finland OY, Vantaa, Finland) on both eyes, and VD and PD was assessed using OCTA (Spectral-Domain Cirrus HD-OCT 5000, Carl Zeiss Meditec, Dublin, USA) on both eyes, immediately before and immediately after the sub-Tenon anesthesia was applied on the eye planned to receive same day cataract surgery. The contralateral eye was examined to serve as control to strengthen the robustness of the comparative analysis. After cataract surgery no further images were taken (Figure 1).

As described in previous studies, for the sub-Tenon anesthesia, the ocular surface was initially anesthetized with Tetracaine 1% eye drops, the area around the eye was thoroughly cleaned, and the ocular surface was disinfected with a 5% povidone-iodine solution for 90 s (Enz et al., 2022). Patients were positioned to look upwards and laterally, and a minor incision was made into the conjunctiva and Tenon’s capsule at the inferomedial quadrant. The Tenons tissue was gently dissected from the sclera using a blunt scissor. A syringe with a blunt cannula was then carefully advanced behind the globe’s equator. Two ml of preservative free mepivacaine 2% with additives solution, sufficient amount for analgesia (Tsatsos et al., 2011), were injected into the space between the sclera and Tenon in proximity to the posterior pole, while care was taken to avoid excessive pressure on the globe. Mepivacaine is considered to have the least pronounced vaso-constrictive effect (Sung et al., 2012; Lindorf, 1979).

Immediately after the application of the anesthesia all above-described measurements were re-assessed on both eyes. Subsequently, to ensure that the same retinal region examined was assessed pre- and post-interventionally, the follow-up OCTA imaging modality was employed. During imaging, the investigators assessed the signal strength index (SSI), as well as the quality of the images regarding image clarity, media opacities, ocular saccades, and presence of blink or other artifacts. Only the best images were selected for postprocessing. In few cases either no OCTA image at all or only of very low quality could be captured post-interventionally. In these cases, the patient was excluded from the study. Subsequently the patient was prepped again for the planned cataract surgery.

### OCTA analysis

All scans were analyzed using Cirrus OCTA software (AngioPlex Metrix version 11.0). The OCTA scans (3x3mm or 6x6mm) were centered on the fovea and the superficial capillary plexus (SCP) vessel density (VD) and perfusion density (PD) were assessed automatically in an 1 mm diameter foveal area circle (central), a 3 mm parafoveal area diameter annulus outside the fovea (inner), and the entire macular area (full) according to the ETDRS-style. As described by Guemes-Villahoz et al. (2021) VD is defined as the total length of perfused vasculature per unit area (mm/mm^2^), while PD as the area of perfused vasculature per unit area within the region of interest (%). The data were analyzed to assess changes in the superficial capillary plexus.

### Statistical analysis

The primary outcome was defined as the change in vessel density (VD) and perfusion density (PD) before and after the administration of sub-Tenon anesthesia. Statistical analysis was performed with GraphPad Prism version 10.4.0. Continuous variables are reported as mean ± standard deviation, categorical variables as number. After testing for normal distribution, paired t-test was used to compare pre- and postinterventional changes. *p*-values < 0.05 were considered statistically significant. To analyze correlation between image quality and VD or PD respectively, the Pearson correlation coefficient was calculated using all available measurements (*n* = 80).

## Results

Among the 20 patients included in the study, the mean age was 69.8 ± 10.2 years, with 11 females and 9 males. All patients completed the study without any significant adverse events related to anesthesia.

### OCTA findings

**Figure 2:**
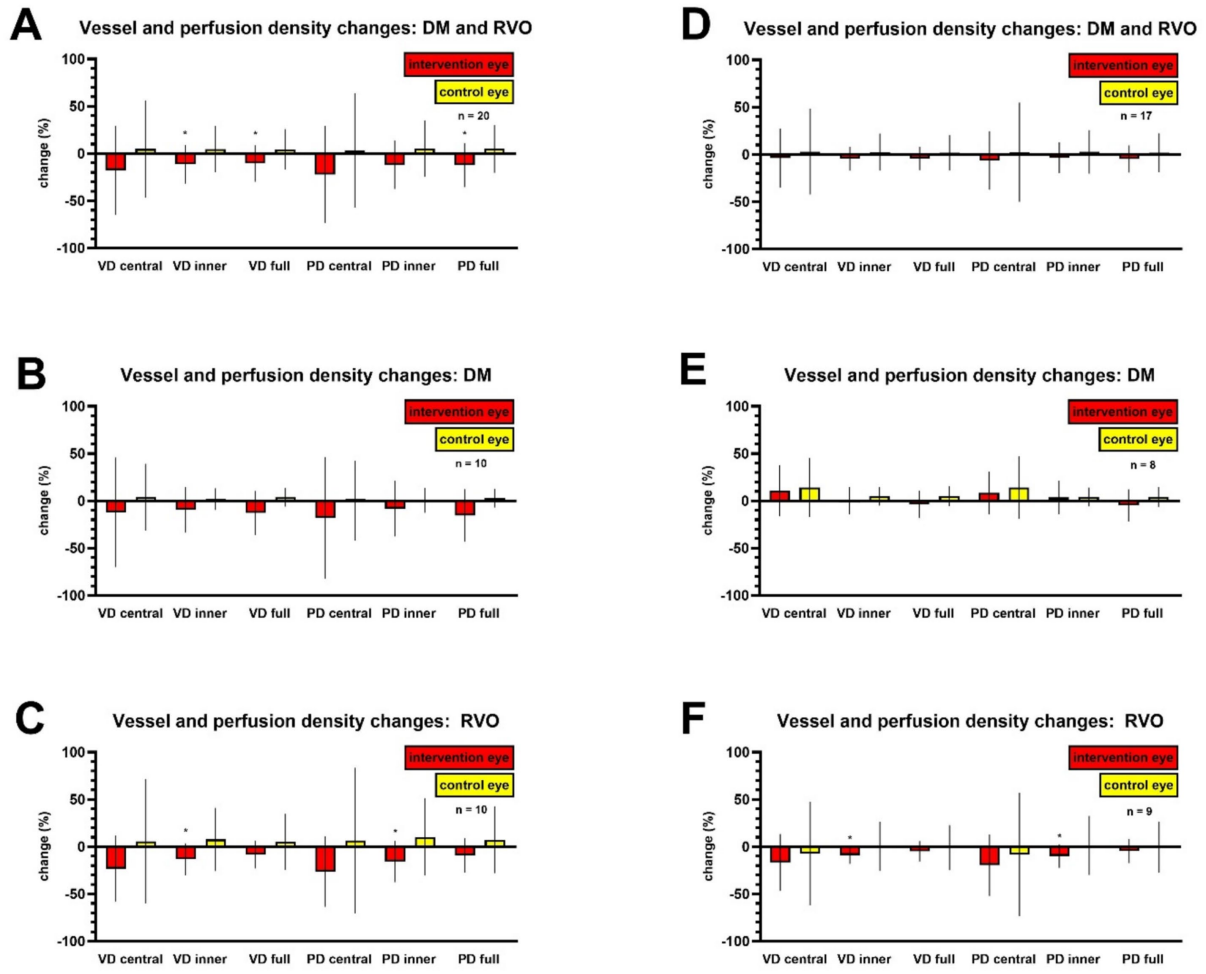
Intervention-related changes before and after the anesthetic injection. Values are presented as mean +/− standard deviation. The symbol (*) indicates the statistically significant changes (*p* < 0.05) from pre- to post-anesthesia measurement. **(A)** Changes observed when the data of all participants were evaluated (*n* = 20). **(B)** Changes observed when the data of all participants with DM were evaluated (*n* = 10). **(C)** Changes observed when the data of all participants with RVO were evaluated (*n* = 10). **(D)** Changes observed when the data of participants with OCTA image quality < 6 were excluded (*n* = 17). **(E)** Changes observed in the DM group when the data of participants with OCTA image quality < 6 were excluded (*n* = 8). **(F)** Changes observed in the RVO group when the data of participants with OCTA image quality < 6 were excluded (*n* = 9). DM = diabetes mellitus, RVO = retinal vein occlusion, VD = vessel density; PD = perfusion density.

Initially, a correlation between sub-Tenon anesthesia and a reduction in retinal perfusion was observed. In the treated eyes, the mean VD in the superficial capillary plexus of the macular area (full) decreased by −1.5 ± 2.8 mm/mm^2^, from 14.4 ± 3.7 mm/mm^2^ pre- anesthesia to 12.8 ± 4.0 mm/mm^2^ post- anesthesia (*p* = 0.02). Similarly, the PD reduced from 30.2 ± 6.4% to 26.5 ± 7.0% which correlates to a reduction of −3.7 ± 7.0% (*p* = 0.03). In the control eyes the mean VD in the superficial capillary plexus of the macular area (full) was reduced by 0.7 ± 3.4 mm/mm^2^ (*p* = 0.36) while the PD reduced by 1.6 ± 8.2% (*p* = 0.38) (Tables 1–3; Figure 2). This difference between treated and control eyes in regard to the VD (*p* = 0.02) as well as the PD (*p* = 0.03) suggested that sub-Tenon anesthesia might lead to temporary hypoperfusion in the retinal microvasculature.

**Table 1:** Descriptive information of patients with diabetes mellitus (*n* = 10).

Diabetes mellitus	Pre intervention	Post intervention	*p*-value	Change in absolute value	Change in %
Age (years)	63.7 (9.9)				
Sex^#^, female/male	5/5				
Systolic BP (mmHg)	145.2 (37.1)	145.6 (34.9)	0.94	0.4 (17.1)	0.3
Diastolic BP (mmHg)	87.7 (18.7)	82.4 (15.2)	0.32	−5.3 (15.9)	−6.0
Intervention eye					
IOP (mmHg)	18.2 (4.9)	17.6 (4.9)	0.67	−0.4 (2.9)	−2.2
Time to intervention (min)	11.5 (6.8)				
Time after intervention (min)		9.1 (7.1)			
Quality of OCTA	8.4 (1.5)	7.6 (2.1)	0.10	−0.8 (1.4)	−9.5
VD (mm/mm^2^) central	8.3 (4.3)	7.4 (2.8)	0.53	−0.8 (4.2)	−9.6
VD (mm/mm^2^) inner	15.2 (4.5)	14.8 (4.6)	0.25	−1.4 (3.7)	−9.2
VD (mm/mm^2^) full	15.9 (3.5)	14.1 (3.9)	0.12	−1.8 (3.4)	−11.3
PD (%) central	16.7 (9.1)	14.0 (5.0)	0.40	−2.7 (9.6)	−16.2
PD (%) inner	32.2 (7.5)	29.7 (8.2)	0.41	−2.5 (9.3)	−7.8
PD (%) full	33.3 (6.6)	28.4 (6.3)	0.11	−4.7 (8.4)	−14.1
Control eye					
IOP (mmHg)	17.7 (5.5)	18.7 (5.3)	0.25	1.0 (2.5)	5.6
Time to intervention (min)	11.0 (7.2)				
Time after intervention (min)		11.4 (6.5)			
Quality of OCTA	8.9 (1.4)	9.2 (1.0)	0.40	0.3 (1.1)	3.4
VD (mm/mm^2^) central	9.1 (3.8)	9.4 (3.6)	0.73	0.4 (3.1)	4.4
VD (mm/mm^2^) inner	16.4 (3.8)	16.8 (3.2)	0.62	0.3 (1.9)	1.8
VD (mm/mm^2^) full	15.5 (3.5)	16.1 (2.8)	0.25	0.6 (1.5)	3.9
PD (%) central	19.1 (12.4)	19.1 (9.6)	>0.99	0.0 (7.5)	0.0
PD (%) inner	34.1 (6.5)	34.3 (5.2)	0.90	0.2 (4.4)	0.6
PD (%) full	32.2 (6.1)	33.1 (4.0)	0.38	0.9 (7.2)	2.8

Values are presented as mean (standard deviation), except for variables marked with #, which are reported as counts. *p*-values were calculated using the paired t-test. IOP = intraocular pressure; BP = blood pressure; OCTA = Optical Coherence Tomography Angiography; VD = vessel density; PD = perfusion density; Change (absolute) = numerical change from pre- to postintervention (in the original units of the variable), Change (%) = the relative change of the mean from pre- to postintervention, expressed as a percentage.

**Table 2:** Descriptive characteristics of patients with retinal vein occlusion (*n* = 10).

Retinal vein occlusion	Pre intervention	Post intervention	*p*-value	Change in absolute value	Change in %
Age (years)	76.0 (6.1)				
Sex^#^, female/male	6/4				
Systolic BP (mmHg)	167.9 (21.2)	160.1 (21.1)	**0.05**	−7.8 (10.8)	−4.6
Diastolic BP (mmHg)	85.6 (13.8)	91.7 (10.9)	0.16	6.1 (12.4)	7.1
Intervention eye					
IOP (mmHg)	17.5 (5.3)	18.3 (4.1)	0.51	0.9 (3.8)	5.1
Time to intervention (min)	10.2 (5.4)				
Time after intervention (min)		6.2 (3.1)			
Quality of OCTA	7.9 (1.4)	7.4 (1.8)	0.14	−0.5 (0.97)	−6.3
VD (mm/mm^2^) central	6.3 (3.5)	4.7 (3.6)	0.07	−1.7 (2.6)	−27.0
VD (mm/mm^2^) inner	14.3 (3.4)	12.2 (4.3)	**0.03**	−2.0 (2.6)	−14.0
VD (mm/mm^2^) full	12.8 (3.3)	11.6 (4.0)	0.11	−1.2 (2.1)	−9.4
PD (%) central	13.0 (7.9)	9.1 (7.1)	0.05	−3.9 (5.5)	−30.0
PD (%) inner	30.7 (5.9)	25.7 (8.0)	**0.05**	−4.9 (6.9)	−16.0
PD (%) full	27.3 (5.0)	24.6 (7.4)	0.15	−2.7 (5.6)	−9.9
Control eye					
IOP (mmHg)	16.2 (3.4)	15.3 (3.0)	0.31	−0.9 (2.7)	−5.6
Time to intervention (min)	11.3 (7.9)				
Time after intervention (min)		9.5 (3.4)			
Quality of OCTA	8.5 (1.7)	8.7 (1.2)	0.73	0.2 (1.8)	2.4
VD (mm/mm^2^) central	8.5 (4.5)	9.0 (3.2)	0.79	0.5 (5.8)	5.9
VD (mm/mm^2^) inner	16.4 (5.5)	17.6 (3.5)	0.49	1.3 (5.5)	7.9
VD (mm/mm^2^) full	15.9 (4.7)	16.7 (3.3)	0.60	0.8 (4.6)	5.0
PD (%) central	16.5 (9.9)	17.7 (7.0)	0.79	1.2 (13.8)	7.3
PD (%) inner	33.1 (9.8)	36.5 (6.9)	0.44	3.5 (13.7)	10.6
PD (%) full	32.6 (8.3)	35.0 (6.9)	0.53	2.4 (11.4)	7.4

Values are presented as mean (standard deviation), except for variables marked with #, which are reported as counts. *p*-values were calculated using the paired t-test. IOP = intraocular pressure; BP = blood pressure; OCTA = Optical Coherence Tomography Angiography; VD = vessel density; PD = perfusion density; Change (absolute) = numerical change from pre- to postintervention (in the original units of the variable), Change (%) = the relative change of the mean from pre- to postintervention, expressed as a percentage. Bold values indicate statistically significant results (*p* < 0.05).

**Table 3:** Descriptive information of all patients (*n* = 20).

All patients	Pre intervention	Post intervention	*p*-value	Change in absolute value	Change in %
Age (years)	69.8 (10.2)				
Sex^#^, female/male	11/9				
Systolic BP (mmHg)	156.6 (31.6)	152.9 (29.0)	0.27	−3.7 (14.5)	−2.4
Diastolic BP (mmHg)	86.7 (16.0)	87.1 (13.7)	0.91	0.4 (15.1)	0.5
Intervention eye					
IOP (mmHg)	17.8 (5.0)	18.0 (4.4)	0.76	0.2 (3.3)	1.1
Time to intervention (min)	10.9 (6.0)				
Time after intervention (min)		7.7 (5.5)			
Quality of OCTA	8.2 (1.4)	7.5 (1.9)	**0.02**	−0.7 (1.2)	−8.5
VD (mm/mm^2^) central	7.3 (3.9)	6.0 (3.4)	0.11	−1.3 (3.4)	−17.8
VD (mm/mm^2^) inner	15.2 (4.0)	13.5 (4.5)	**0.02**	−1.7 (3.1)	11.2
VD (mm/mm^2^) full	14.4 (3.7)	12.8 (4.0)	**0.02**	−1.5 (2.8)	−10.4
PD (%) central	14.9 (8.3)	11.6 (6.5)	0.07	−3.3 (7.6)	−22.1
PD (%) inner	31.4 (6.7)	27.7 (8.1)	0.05	−3.7 (8.0)	−11.8
PD (%) full	30.2 (6.4)	26.5 (7.0)	**0.03**	−3.7 (7.0)	−12.3
Control eye					
IOP (mmHg)	17.0 (4.5)	17.0 (4.5)	0.97	0.0 (2.7)	0.0
Time to intervention (min)	11.2 (7.4)				
Time after intervention (min)		10.5 (5.1)			
Quality of OCTA	8.7 (1.5)	9.0 (1.1)	0.44	0.3 (1.4)	3.4
VD (mm/mm^2^) central	8.8 (4.1)	9.2 (3.3)	0.67	0.4 (4.5)	4.5
VD (mm/mm^2^) inner	16.4 (4.6)	17.2 (3.3)	0.39	0.8 (4.0)	4.9
VD (mm/mm^2^) full	15.7 (4.1)	16.4 (3.0)	0.36	0.7 (3.4)	4.5
PD (%) central	17.8 (11.0)	18.4 (8.2)	0.81	0.6 (10.8)	3.4
PD (%) inner	33.6 (8.1)	35.4 (5.7)	0.42	1.8 (10.0)	5.4
PD (%) full	32.4 (7.1)	34.0 (5.6)	0.38	1.6 (8.2)	4.9

Values are presented as mean (standard deviation), except for variables marked with #, which are reported as counts. *p*-values were calculated using the paired t-test. IOP = intraocular pressure; BP = blood pressure; OCTA = Optical Coherence Tomography Angiography; VD = vessel density; PD = perfusion density; Change (absolute) = numerical change from pre- to postintervention (in the original units of the variable), Change (%) = the relative change of the mean from pre- to postintervention, expressed as a percentage. Bold values indicate statistically significant results (*p* < 0.05).

However, upon further analysis, it was found that some of the post- anesthesia OCTA images obtained were of low quality - defined as SSI below the manufacturer’s recommended threshold (< 6) (Tables 1–3). Hence, we further investigated whether the poor image quality might be responsible for the postinterventional reduction seen in VD and PD measurements. The Pearson’s correlation coefficient (r) showed a strong correlation between image quality and VD or PD. It was 0.76 (95% CI 0.65–0.84, *p* < 0.0001, *n* = 80) for VD (full) and image quality, and 0.72 (95% CI 0.60–0.81, *p* < 0.0001, *n* = 80) for PD (full) and image quality, respectively. Consequently, as part of a secondary analysis the statistical significance of the change in VD and PD before and after sub-Tenon anesthesia was re-examined after exclusion of lower quality images. Similarly to equivalent studies, such as the one performed from Yao et al., images of quality index below 6 were excluded. In total, 3 images of 3 patients were excluded. In this new analysis, the postinterventional VD and PD reduction were no longer statistically significant (Table 4). Table 3 depicts how OCTA image quality declined in intervention eyes versus controls (−8.5% vs. + 3.4%). In the full cohort, VD inner, VD full, and PD full showed statistically significant reductions (*p* ≈ 0.03) that disappeared after quality filtering, while central metrics were non-significant with high variability. These apparent VD/PD decreases likely reflect image-quality artefacts rather than true perfusion changes. To further assess whether the observed post-intervention OCTA changes persisted after accounting for image-quality–related artefacts (i.e., potential confounding by scan quality), we conducted—as an additional statistical approach—a patient-level difference-in-differences (DiD) sensitivity analysis adjusting for inter-eye and longitudinal differences in OCTA image quality. Using a patient-level DiD regression in GraphPad Prism [DiD (outcome) ~ mean-centered DiD (image quality)], image quality was a strong predictor of OCTA changes (e.g., VD full *β* = 1.733, 95% CI 1.245–2.222; *p* < 0.0001). Importantly, the quality-adjusted DiD effect (intercept at mean quality) was not different from zero (VD full 0.910, 95% CI − 0.458 to 2.277; *p* = 0.179), and results were consistent across VD regions (central *p* = 0.557; inner *p* = 0.301) and perfusion metrics.

**Table 4:** Descriptive information of patients with SSI ≥ 6 (*n* = 17).

Patients with SSI ≥ 6	Pre intervention	Post intervention	*p*-value	Change in absolute value	Change in %
Age (years)	70.8 (9.8)				
Sex^#^, female/male	9/8				
Systolic BP (mmHg)	156.8 (32.6)	154.2 (30.1)	0.51	−2.5 (15.3)	−1.6
Diastolic BP (mmHg)	88.4 (15.4)	87.5 (13.7)	0.79	−0.9 (13.3)	−1.0
Intervention eye					
IOP (mmHg)	18.2 (5.1)	18.2 (4.0)	0.92	0.08 (3.5)	0.4
Time to intervention (min)	10.0 (6.1)				
Time after intervention (min)		7.5 (4.6)			
Quality of OCTA	8.4 (1.5)	8.0 (1.5)	0.16	−0.4 (1.0)	−4.8
VD (mm/mm^2^) central	6.6 (3.5)	6.3 (3.4)	0.63	−0.3 (2.3)	−4.5
VD (mm/mm^2^) inner	15.1 (4.3)	14.4 (4.1)	0.16	−0.7 (1.9)	−4.6
VD (mm/mm^2^) full	14.2 (3.9)	13.6 (3.8)	0.17	−0.61 (1.8)	−4.3
PD (%) central	13.2 (6.9)	12.2 (6.4)	0.41	−0.94 (4.6)	−7.1
PD (%) inner	30.8 (7.1)	29.7 (6.9)	0.38	−1.1 (5.01)	−3.6
PD (%) full	29.4 (6.2)	28.0 (6.3)	0.19	−1.4 (4.3)	−4.8
Control eye					
IOP (mmHg)	17.2 (4.1)	17.4 (4.3)	0.70	0.3 (2.8)	1.7
Time to intervention (min)	10.5 (7.8)				
Time after intervention (min)		10.2 (4.6)			
Quality of OCTA	8.8 (1.5)	8.8 (1.1)	>0.99	0.0 (1.3)	0.0
VD (mm/mm^2^) central	8.3 (3.3)	8.6 (2.8)	0.79	0.26 (4.0)	3.1
VD (mm/mm^2^) inner	16.9 (4.4)	17.3 (3.4)	0.60	0.4 (3.2)	2.4
VD (mm/mm^2^) full	16.0 (4.1)	16.3 (3.2)	0.69	0.3 (2.9)	1.9
PD (%) central	16.2 (7.3)	16.6 (5.5)	0.85	0.4 (9.3)	2.5
PD (%) inner	34.1 (7.3)	35.0 (5.5)	0.63	0.9 (7.7)	2.6
PD (%) full	32.7 (7.0)	33.2 (5.1)	0.74	0.6 (6.6)	1.8

Values are presented as mean (standard deviation), except for variables marked with #, which are reported as counts. *p*-values were calculated using the paired t-test. SSI = signal strength index, IOP = intraocular pressure; BP = blood pressure; OCTA = Optical Coherence Tomography Angiography; VD = vessel density; PD = perfusion density; Change (absolute) = numerical change from pre- to postintervention (in the original units of the variable), Change (%) = the relative change from pre- to postintervention, expressed as a percentage.

### Intraocular pressure and blood pressure

No significant differences were observed in IOP between treated and control eyes before and after sub-Tenon anesthesia (Tables 1–3). The mean IOP in the treated eyes increased by 0.2 ± 3.3 mmHg (mean ± SD) (*p* = 0.76), while in the control eyes, the increase was 0.0 ± 2.7 mmHg (mean ± SD) (*p* = 0.97). Similarly, BP changes from baseline were minimal, with a mean change of −3.7 ± 14.5 mmHg in the systolic BP (change −2.4%) and 0.4 ± 15.1 mmHg in the diastolic BP (change 0.5%) (*p* = 0.27 and 0.91 respectively). However, when the RVO subpopulation was analyzed separately, a slight statistically significant increase in systolic BP was observed after the anesthesia injection (*p* = 0.05).

## Discussion

Even though sub- Tenon anesthesia is regarded as a safe and favored procedure, some cases of retinal ischemia have been reported over the years. Possible explanations of this complication are related to an increase in IOP, formation of a retrobulbar hemorrhage, or compression of blood vessel, while patients with compromised retinal perfusion or significant cardiovascular risk factors are considered high-risk (Kumar et al., 2011). Transient or permanent central retinal artery occlusions because of sub-Tenons anesthesia have been described in 1982 by Klein et al. (1982), in 2003 by Feibel and Guyton (2003), and in 2008 by Fry et al. Fry and Ring (2008). These occlusions were observed even in the absence of orbital hemorrhage, and a spasm of the central retinal artery—either pharmacologically induced or due to a compressive effect—was suspected (Feibel and Guyton, 2003). In contrast to the current study, four patients described by Klein et al. were diagnosed with either a severe hematologic disorder, including sickle cell retinopathy in two cases, or a vascular pathology, which encompassed diabetic retinopathy, carotid artery insufficiency associated with ocular ischemia, and neovascular glaucoma.

The initial results of the current study appeared to show a reduction of both VD and PD following anesthesia, which could have confirmed previous concerns about the hemodynamic impact of this technique (Coşkun et al., 2014; Enz et al., 2022). However, upon closer examination, the reduction in VD and PD was found to be associated with lower-quality OCTA images, which were likely a result of the intervention related artifacts such as surface irregularities caused by Betadine application, dry eye, instable tear film, presence of blood or mucus and debris around the injection site. A previous OCTA study examining the effects of sub-Tenon anesthesia similarly described a greater signal reduction in interventional than in controls eyes (Enz et al., 2022), while Czakó et al. (2019) and other researchers have already emphasized the influence of images quality on OCTA metrics and that it can potentially lead to misinterpretations. In the current study, once 3 low quality images were excluded, no significant reduction in VD or PD was any longer observed. What further supported that that the observed VD/PD reductions reflect measurement artefact rather than a true hemodynamic effect is that the Quality-adjusted DiD regression demonstrated that the apparent OCTA changes were largely driven by image-quality variation and were no longer present after adjustment.

The manifestation of OCTA artifacts during picture acquisition is a common and known challenge. A variety of studies has reported that the prevalence of OCTA artifacts can be up 89.4–97% (Holmen et al., 2020). Such artifacts can impair the reliability of the OCTA analysis since they might potentially misrepresent blood vessels and their morphology, mask certain pathologies in the vascular plexus of the retina and hereby lead to misinterpretations. There are different reasons and types of OCTA artifacts. Most commonly seen are motion artifacts due to saccades, blinking, or head motion that lead to disruptions in the image’s continuity despite eye-tracking mechanisms (Lauermann et al., 2018); further common artifacts appear due to signal attenuation factors usually as a result of an element obstructing the light transmission (such as cataracts, floaters, debris in the tear film) (Anvari et al., 2021); and finally segmentation errors (Lauermann et al., 2018). However, the presence of OCTA artifacts does not necessarily result in the total exclusion of the image. When the images are manually controlled and evaluated, to assess the extent and the potential scale of the artifact’s impact, they can still be eligible for further analysis (Holmen et al., 2020). For this purpose, in this study for each eye multiple images were taken, those with the least relevant artifacts and the best quality were further analyzed, in cases where no good images could be obtained for either the intervention or the control eye the patient was totally excluded from the study.

Our study found no evidence of negative hemodynamic effects on the retinal microvasculature in patients with pre-existing perfusion deficits, which is particularly reassuring for ophthalmic surgeons who favor sub-Tenon anesthesia for ocular procedures. The absence of significant changes in IOP or systemic BP further supports the safety of sub-Tenon anesthesia, even in patients with compromised retinal microvasculature. Our findings are consistent with the broader literature indicating that, sub-Tenon anesthesia has minimal or no impact on IOP (Enz et al., 2022; Pianka et al., 2001; Alwitry et al., 2001). To our knowledge this is the first study examining the effects of sub-Tenon anesthesia on eyes with compromised retinal perfusion. In addition, our study highlights the importance of image quality when interpreting OCTA results. Artifacts can lead to false-positive findings, which could be mistakenly attributed to real physiological changes.

Despite being a prospective study, we acknowledge several limitations and shortcomings. A principal limitation is the modest sample size, despite the extended recruitment period. This might have reduced the precision and have limited the power of the study to detect small perfusion changes. Recruitment was constrained by the additional testing burden, which impacted theatre workflows, and by the high appointment load commonly faced by patients with DM and RVO (e.g., frequent intravitreal injections), reducing willingness to undergo extra imaging. Furthermore, several initially enrolled patients were excluded because high-quality, artefact-free OCTA images—particularly in the intervention eye—could not be obtained. A further limitation of this study was that the type of cataract and the degree of nuclear sclerosis were not systematically documented. As these parameters are not routinely assessed or recorded in our clinical practice, they were unavailable for analysis. Consequently, it was not possible to evaluate the influence of specific cataract characteristics on image quality, including OCTA signal strength and overall image reliability.

Furthermore, glaucoma—particularly normal-tension glaucoma—was not an exclusion criterion. This may represent an additional limitation, as these patients may exhibit reduced perfusion of end organs, including the optic nerve head and retinal microvasculature. Additionally, the scan dimensions were not standardized in our OCTA protocol. Therefore, some investigators acquired 3 × 3 mm field of view images, while others used 6 × 6 mm fovea-centered scans. This inconsistency was only identified after study completion during data evaluation. In total 7 patients received a 6 × 6 mm field of view measurement, two of which were later on excluded from the study due to too low image quality. For 6 × 6 mm scans, analysis was restricted to the central 3 mm (perifoveal 3–6 mm ring excluded) to improve comparability with 3 × 3 mm data. As 3 × 3 mm OCTA generally affords higher spatial resolution and sensitivity for detecting microvascular changes (Ho et al., 2019), the 6 × 6 mm subset may have reduced spatial sensitivity. Notably, each fellow control eye was acquired with the same scan size as its intervention eye, maintaining within-subject comparability. Patients with glaucoma and the OCTA scan field size have been identified in the raw dataset provided in the Supplementary material. Further, although imaging was performed immediately pre-block and again within minutes post-block to capture acute vasomotor/pressure effects, the post-intervention scans were acquired at 7.7 ± 5.5 min, which may have coincided with time-dependent normalization of intraocular pressure and perfusion. Finally, some eyes in both the DM and RVO groups had macular edema at the time of cataract surgery and, consequently, during the study imaging. This factor was not analyzed separately, which may have influenced our results.

## Conclusion

In conclusion, our study did not identify any significant reduction in VD or PD following sub-Tenon anesthesia when accounting for the OCTA image quality. These findings support the further use of sub-Tenon anesthesia even in patients with retinal perfusion deficits such as those with DM and RVO. Additionally, the study highlights vulnerability of OCTA metrics immediately after periocular anesthesia. Larger-scale research and optimized imaging techniques may help provide more insight into any potential subtle hemodynamic effects of this anesthesia technique.

## Data Availability

The raw data supporting the conclusions of this article will be made available by the authors, without undue reservation.

## References

[ref1] Adekola O. Aribaba O. Musa K. Olatosi J. Asiyanbi G. Rotimi-Samuel A. . (2018). Regional anesthesia for small incision cataract surgery: comparison of subtenon and peribulbar block. J Clin Sci. 15:1. doi: 10.4103/jcls.jcls_5_17

[ref2] Alwitry A. Koshy Z. Browning A. C. Kiel W. Holden R. (2001). The effect of sub-tenon’s anaesthesia on intraocular pressure. Eye (Lond.) 15, 733–735. doi: 10.1038/eye.2001.239, 11826992

[ref3] Antony R. M. Kamath A. R. Jeganathan S. Rodrigues G. R. (2022). A comparison of sub-tenon block with peribulbar block in small-incision cataract surgery. Indian J. Ophthalmol. 70, 3840–3843. doi: 10.4103/ijo.IJO_1553_22, 36308108 PMC9907232

[ref4] Anvari P. Ashrafkhorasani M. Habibi A. Ghasemi F. K. (2021). Artifacts in optical coherence tomography angiography. J. Ophthalmic Vis. Res. 16:271. doi: 10.18502/jovr.v16i2.9091 34055264 PMC8126744

[ref5] Chen F. K. (2017). Day one ‘patch-off’ visual loss due to retinal ischaemic injury: can we blame sub-tenon’s or peribulbar anaesthesia? Clin. Experiment. Ophthalmol. 45, 565–567. doi: 10.1111/ceo.13010, 28840664

[ref6] Chua J. Tan B. Wong D. Garhöfer G. Liew X. W. Popa-Cherecheanu A. . (2024). Optical coherence tomography angiography of the retina and choroid in systemic diseases. Prog. Retin. Eye Res. 103:101292. doi: 10.1016/j.preteyeres.2024.101292 39218142

[ref7] Coşkun M. Dağlıoğlu M. C. Davran R. İlhan N. İlhan Ö. Ayhan Tuzcu E. . (2014). Effects of sub-tenon’s anaesthesia on ocular hemodynamics. Can. J. Ophthalmol. 49, 141–144. doi: 10.1016/j.jcjo.2013.10.004, 24767218

[ref8] Creese K. Ong D. Sandhu S. S. Ware D. Harper C. A. Al-Qureshi S. H. . (2017). Paracentral acute middle maculopathy as a finding in patients with severe vision loss following phacoemulsification cataract surgery. Clin. Experiment. Ophthalmol. 45, 598–605. doi: 10.1111/ceo.12945, 28295944

[ref9] Czakó C. István L. Ecsedy M. Récsán Z. Sándor G. Benyó F. . (2019). The effect of image quality on the reliability of OCT angiography measurements in patients with diabetes. Int J Retina Vitr. 5:46. doi: 10.1186/s40942-019-0197-4 PMC6829984 31709114

[ref10] Ellis P. P. (1964). Induced spasm in the retinal arterioles of cats: II. Influences of physical factors and drugs. Arch. Ophthalmol. 71, 706–711. doi: 10.1001/archopht.1964.00970010722020, 14120664

[ref11] Enz T. J. Maloca P. M. Tschopp M. Menke M. N. Tribble J. R. Williams P. A. . (2022). Volume-rendered optical coherence tomography angiography during ocular interventions: advocating for noninvasive intraoperative retinal perfusion monitoring. J. Biophotonics 15:e202200169. doi: 10.1002/jbio.202200169 36089335

[ref12] Feibel R. M. Guyton D. L. (2003). Transient central retinal artery occlusion after posterior sub-tenon’s anesthesia. J Cataract Refract Surg 29, 1821–1824. doi: 10.1016/s0886-3350(02)01975-2, 14522307

[ref13] Fry R. A. Ring P. (2008). Cilioretinal artery occlusion associated with sub-tenon’s regional blockade. Clin. Experiment. Ophthalmol. 36, 196–197. doi: 10.1111/j.1442-9071.2008.01686.x, 18352889

[ref14] Gandhi S. Pattathil N. Choudhry N. (2024). OCTA: essential or gimmick? Ophthalmol Ther. 13, 2293–2302. doi: 10.1007/s40123-024-00985-0, 38970762 PMC11341508

[ref15] Guemes-Villahoz N. Burgos-Blasco B. Perez-Garcia P. Fernandez-Vigo J. I. Morales-Fernandez L. Donate-Lopez J. . (2021). Retinal and peripapillary vessel density increase in recovered COVID-19 children by optical coherence tomography angiography. J. AAPOS 25, 325.e1–325.e6. doi: 10.1016/j.jaapos.2021.06.004, 34687877 PMC8527103

[ref16] Guise P. (2012). Sub-tenon’s anesthesia: an update. Local Reg Anesth. 5, 35–46. doi: 10.2147/LRA.S16314, 22915900 PMC3417980

[ref17] Haydinger C. D. Ferreira L. B. Williams K. A. Smith J. R. (2023). Mechanisms of macular edema. Front Med (Lausanne). 10:1128811. doi: 10.3389/fmed.2023.1128811 36960343 PMC10027768

[ref18] Ho J. Dans K. You Q. Nudleman E. D. Freeman W. R. (2019). Comparison of 3 mm × 3 mm versus 6 mm × 6 mm optical coherence tomography angiography scan sizes in the evaluation of non–proliferative diabetic retinopathy. Retina 39, 259–264. doi: 10.1097/IAE.0000000000001951, 29190249 PMC5963959

[ref19] Holmen I. C. Konda S. M. Pak J. W. McDaniel K. W. Blodi B. Stepien K. E. . (2020). Prevalence and severity of artifacts in optical coherence tomographic angiograms. JAMA Ophthalmol. 138:119. doi: 10.1001/jamaophthalmol.2019.4971 31804666 PMC6902206

[ref20] Hulbert M. F. Yang Y. C. Pennefather P. M. Moore J. K. (1998). Pulsatile ocular blood flow and intraocular pressure during retrobulbar injection of lignocaine: influence of additives. J. Glaucoma 7, 413–416, 9871864

[ref21] Klein M. L. Jampol L. M. Condon P. I. Rice T. A. Serjeant G. R. (1982). Central retinal artery occlusion without retrobulbar hemorrhage after retrobulbar anesthesia. Am. J. Ophthalmol. 93, 573–577, 7081356 10.1016/s0002-9394(14)77371-4

[ref22] Kohner E. M. Patel V. Rassam S. M. B. (1995). Role of blood flow and impaired autoregulation in the pathogenesis of diabetic retinopathy. Diabetes 44, 603–607, 7789621 10.2337/diab.44.6.603

[ref23] Kumar C. M. Dodds C. (2006). Sub-tenon’s anesthesia. Ophthalmol. Clin. N. Am. 19, 209–219. doi: 10.1016/j.ohc.2006.02.008 16701158

[ref24] Kumar C. M. Eid H. Dodds C. (2011). Sub-tenon’s anaesthesia: complications and their prevention. Eye (Lond.) 25, 694–703. doi: 10.1038/eye.2011.69, 21455245 PMC3178142

[ref25] Kumar C. M. Williamson S. Manickam B. (2005). A review of sub-tenon’s block: current practice and recent development. Eur. J. Anaesthesiol. 22, 567–577. doi: 10.1017/s0265021505000967, 16119592

[ref26] Kwiterovich K. A. Maguire M. G. Murphy R. P. Schachat A. P. Bressler N. M. Bressler S. B. . (1991). Frequency of adverse systemic reactions after fluorescein angiography. Ophthalmology 98, 1139–1142, 1891225 10.1016/s0161-6420(91)32165-1

[ref27] Lauermann J. L. Woetzel A. K. Treder M. Alnawaiseh M. Clemens C. R. Eter N. . (2018). Prevalences of segmentation errors and motion artifacts in OCT-angiography differ among retinal diseases. Graefes Arch. Clin. Exp. Ophthalmol. 256, 1807–1816. doi: 10.1007/s00417-018-4053-2 29982897

[ref28] Lindorf H. H. (1979). Investigation of the vascular effect of newer local anesthetics and vasoconstrictors. Oral Surg. Oral Med. Oral Pathol. 48, 292–297. doi: 10.1016/0030-4220(79)90026-4, 291855

[ref29] Meyer P. Flammer J. Lüscher T. F. (1993). Local anesthetic drugs reduce endothelium-dependent relaxations of porcine ciliary arteries. Invest. Ophthalmol. Vis. Sci. 34, 2730–2736, 8344795

[ref30] O’Day R. Harper C. A. Wickremasinghe S. S. (2019). Central retinal artery occlusion showing features of paracentral acute middle maculopathy following uncomplicated pterygium surgery. Clin. Experiment. Ophthalmol. 47, 141–143. doi: 10.1111/ceo.13353, 29962106

[ref31] Parkar T. Gogate P. Deshpande M. Adenwala A. Maske A. Verappa K. (2005). Comparison of subtenon anaesthesia with peribulbar anaesthesia for manual small incision cataract surgery. Indian J. Ophthalmol. 53, 255–259. doi: 10.4103/0301-4738.18907, 16333174

[ref32] Pianka P. Weintraub-Padova H. Lazar M. Geyer O. (2001). Effect of sub-tenon’s and peribulbar anesthesia on intraocular pressure and ocular pulse amplitude. J Cataract Refract Surg 27, 1221–1226. doi: 10.1016/s0886-3350(01)00797-0, 11524193

[ref33] Rüschen H. Bremner F. D. Carr C. (2003). Complications after sub-tenon’s eye block. Anesth. Analg. 96:273. doi: 10.1097/00000539-200301000-00054, 12505965

[ref34] Stevens J. D. (1992). A new local anesthesia technique for cataract extraction by one quadrant sub-tenon’s infiltration. Br. J. Ophthalmol. 76, 670–674. doi: 10.1136/bjo.76.11.670, 1477043 PMC504372

[ref35] Sung H. J. Ok S. H. Sohn J. Y. Son Y. H. Kim J. K. Lee S. H. . (2012). Vasoconstriction potency induced by aminoamide local anesthetics correlates with lipid solubility. J. Biomed. Biotechnol. 2012, 1–7. doi: 10.1155/2012/170958 22778542 PMC3385964

[ref36] Tsatsos M. Burnett C. A. Broadway D. C. Eke T. (2011). Local anaesthesia for trans-scleral cyclodiode laser procedures: surgeon and patient satisfaction with sub-tenon’s and peribulbar anaesthesia. Clin. Experiment. Ophthalmol. 39, 472–473. doi: 10.1111/j.1442-9071.2010.02483.x, 21176042

[ref37] Watkins R. Beigi B. Yates M. Chang B. Linardos E. (2001). Intraocular pressure and pulsatile ocular blood flow after retrobulbar and peribulbar anaesthesia. Br. J. Ophthalmol. 85, 796–798. doi: 10.1136/bjo.85.7.796, 11423451 PMC1724026

[ref38] Yannuzzi L. A. Slakter J. S. Sorenson J. A. Guyer D. R. Orlock D. A. (2012). Digital indocyanine green videoangiography and choroidal neovascularization. Retina 32:191. doi: 10.1097/IAE.0b013e31823f98c7 1384094

