# Supplementary figures and images for: Retinal hemodynamic effects of sub-Tenon anesthesia
Source: Front Neurosci. 2026 Feb 27;20:1759889. doi: 10.3389/fnins.2026.1759889 (PMC12982183; doi:10.3389/fnins.2026.1759889)

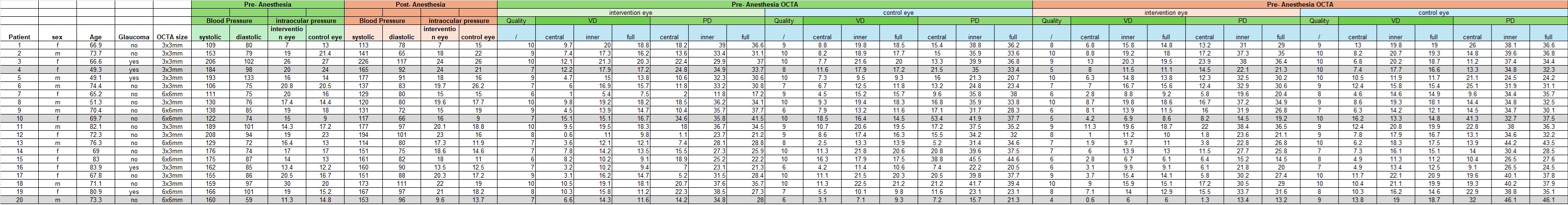

Supplement: Supplementary file 1 [file Image_1.JPEG]
